# Experimental Investigations Regarding the Structural Damage Monitoring of Strands Wire Rope within Mechanical Systems

**DOI:** 10.3390/ma13153439

**Published:** 2020-08-04

**Authors:** Carmen Debeleac, Silviu Nastac, Gina Diana Musca (Anghelache)

**Affiliations:** Engineering and Agronomy Faculty in Braila, Research Center for Mechanics of Machines and Technological Equipment, “Dunarea de Jos” University of Galati, 810017 Braila, Romania; danghelache@ugal.ro

**Keywords:** multi-wire strands rope, experimental transitory vibrating regime, joint time-frequency analysis, high-order spectral analysis, wavelet, structural health monitoring (SHM), non-destructive testing, damage diagnostics

## Abstract

This paper deals with the area of structural damage monitoring of steel strands wire ropes embedded into various equipment and mechanical systems. Of the currently available techniques and methods for wire ropes health monitoring, the authors focused on the group of techniques based on operational dynamics investigation of such systems. Beyond the capability and efficiency of both occasionally and continuously monitoring application, the dynamics-based methods are able to provide additional information regarding the structural integrity and functional operability of the entire ensemble embedding the wire ropes. This paper presents the results gained by the authors using a laboratory setup that can simulate the operational condition usually used for regular applications of wire ropes. The investigations were conducted on three directions of acquired signals post-processing. Firstly, the classical fast Fourier transform was used to evaluate the potential changes within the spectral distribution of transitory response. The other two directions involved high-order spectral analyses in terms of bi-spectrum and Wigner–Ville distribution and multi-scale analysis based methods such as complex wavelet cross-correlation and complex wavelet coherency. The results indicate that each direction of analysis can provide suitable information regarding potential wire rope damage, but the ensemble of post-processing methods offers supplementary precision.

## 1. Introduction

The magnetic rope testing method is one of the common ways of damage detection for steel wire ropes. This is a non-destructive method, having the advantage of a serviceable methodology operating in the frame of available European regulations [1]. This method usage is restricted to steel wire ropes, and it is able to provide useful information related to multiple cracks due to fretting fatigue on peculiar locked coil structure ropes.

One other direction for non-destructive testing and damage detection in cables and wire ropes is represented by the investigations on guided wave propagation phenomena. Hereby, in [2], the authors analyzed the ultrasonic guided waves with application on SHM of multi-wire cables, because of their advantages, including requires a single measurement, large coverage of the acoustic field, and long-range propagation capability. This method presents some difficulties related to the propagation behaviors of guided waves and signal analyses, due to the mechanical coupling of multi-wire ensembles. This was the motivation of the authors to investigate the ultrasonic guided waves propagation in such waveguides. It should be noted that this study contains theoretical, computational, and experimental approaches regarding this problem.

Another interesting study is presented in [3], where, beyond the advantages of guided ultrasonic waves for SHM applications, the authors treated the dispersion curves as a main tool intended for deployment of any guided wave application. An experimental based technique was presented, which provides rapid generation of dispersion curves, in the presence of unknown properties for both material and inspected structure. It should be underlined that the proposed method uses a single source and two acquisition points, thus is experimentally simple to use. The method was validated using synthesized signals, finite element simulations, and experimental tests, with the final accuracy of 1% of the velocity. The authors supposed that the relative error in the calculated velocity due to misplacement is the same as the relative distance error, due to the linearity of velocity–distance relationship [3]. Thus, the reported accuracy has results for 1 mm error of transducer position and 100 mm spacing [3].

A numerical investigation, related to elastic guided waves propagation within a single helical wire, in terms of dispersion curves, was proposed by Treyssède [4]. The finite element method was used to study the wave propagation inside periodic structures, and a new method that avoids the tedious writing of equilibrium equations in a curvilinear coordinate system was developed. The author compared numerical results with the Pochhammer–Chree solutions for infinite isotropic cylinder, in order to evaluate the convergence and accuracy. In addition, for low frequency range, the results were validated with a helical Timoshenko beam model.

Baltazar et al. [5] proposed an interesting experimental based analysis. They considered multi-wire aluminum conductor steel reinforced cable and analyzed the guided waves propagation using time–frequency techniques. A notch, made transversal to the pulse propagation, was used to artificially damage the cable. They concluded that a conversion of longitudinal to flexural modes appeared due to the mechanical contact among the wires. These findings demonstrate that it is difficult to carry out an analysis based on longitudinal or transversal mode identification exclusively.

Kwun and Bartels [6] presented some experimental investigations and observations related to elastic wave dispersion within bounded solids of various configurations. They used an efficient technique consisting of a short elastic wave pulse transmission into the inspected material, with detection of transient waveforms of the transmitted wave, with the help of two non-contacting magnetostrictive devices, followed by classical joint time–frequency analysis of acquired signal. These studies were performed for a large group of solids, between them being a multi-wire rope, and the results were discussed in the frame of available theory.

Schaal et al. [7] considered a structural health investigation algorithm, based on the Hilbert transform applied on the acquired signals from induced guided ultrasonic waves. A practical application on damage detection in multi-wire rope was presented. The proposed algorithm evaluates the time-of-flight of the wave packets, and it can distinguish between the packets from different waves independently. The wave amplitude was also considered as a practical method for damage identification. Note that the method was tested on laboratory setup for single- and multi-wire ropes.

According to the above-mentioned studies, theoretical, computational, and experimental, any of the proposed approaches can be assessed by using proper post-processing techniques applied onto the acquired guided wave signals. Thus, an interesting and useful research was proposed by Wilcox [8]. Starting from the hypotheses that the pulse of energy will spread out in space and time if a guided wave mode is dispersive, which constrains the choice of operating point for a long-range application, the author proposed a signal processing method, which enables the relaxation of this constraint. The author of [8] reported the theoretical basics and an efficient numerical implementation of this method. In addition, an application to experimental signals was provided, in which the improvement in spatial resolution was demonstrated.

Another efficient possibility of recorded signals post-processing, for the case of ultrasonic guided waves was presented in [9]. The basic idea is an entropy-based method to identify the small changes within non-stationary acquired signals. The authors supposed that propagated waves are composed of an infinite number of dispersive vibration modes, which implies difficulties on their analysis. Their method was based on the discrete wavelet transform and the concept of the entropy evolution of non-stationary signals. The analyses were performed using an experimental system, for single- and multi-wire ropes, and the results provide good correlation between the entropy value and level of damage within the wire rope. A high level of correlation was observed even for single-wire damage or condition changes of mechanical contact among the wires. It has to be noted that the proposed method provides low sensitivity to the noise within the signals.

This brief state of the art regarding wire ropes condition monitoring cannot be ended without mentioning some relevant review articles. Firstly, the review article of Schlanbusch et al. [10] refers to the condition monitoring for steel wire ropes. The authors studied offshore wire ropes, firstly presenting the main sources of degradation that typically affect the functionality and, sometimes, accelerate the failure of these ropes. After this introduction, the available non-destructive testing techniques were surveyed, with underlining the electromagnetic based methods that are widely used in mining and construction fields. The review considers the developments within the last years related to the topics of electromagnetic method, acoustic emissions, ultrasound, X- and γ-rays, fiber optics, optical and thermal vision, and current signature analysis. Each method is thoroughly presented. Discussions are focused on the primary methods’ capabilities as follows: proper detection and localization of broken wire, loss estimation of metallic cross-sectional area, robustness (supposing the rough offshore environment), evaluation of both rope and end fittings, and the ability to perform operational tests and analyses.

The authors of [11] provided a useful overview of non-destructive damage detection studies of wire ropes. They also discussed the core issues within this topic. The damage type is firstly introduced, and its causes are systematically explained. Then, the main non-destructive detection techniques are summarized, including electromagnetic detection method, optical detection method, ultrasonic guided wave detection method, and acoustic emission detection method [11].

Condition monitoring of strands wire ropes clearly benefits from the multiple methods available. The previous mentioned review articles [10,11] thoroughly describe the latest technologies in this area. This briefly introduction mainly contains references to electromagnetic and guided wave propagation-based methods because of their good operability and wide utilization within various fields of engineering applications. The authors do not propose to present a comprehensive review of available literature on this research domain. The goal of this short introduction is to underline the following aspects: (a) the availability of various methods and techniques for damage identification within wire ropes; (b) the studies have mainly focused on localization of potential damages, losing sight of the fact that the damage magnitude counts more than position regarding the wire rope failure that finally imposes cable changing; and (c) the gaps regarding vibration-based investigation methods. Additionally, the authors consider that none of the available methods can provide enough capabilities regarding performing operational investigation in the case that the continuously monitoring mode is required. 

Hereby, within the field of current signature analysis, the operational dynamic regime investigations, followed by proper post-processing procedures, allow continuous providing of useful information related to permanent structural health monitoring, potential damage detection, and failure imminence estimation. This paper proposes a vibration-based technique for damage monitoring within strands wire rope. Application simplicity, reduced set of experiments, continuous mode operating, and operational investigation enabling frame are the advantages of this technique.

This paper presents an experimentally-based approach for a multi-wire steel strands rope (MWSSR) sample with fiber core (FC), together with a group of useful post-processing techniques, to provide accurate data regarding the changes within the current dynamic signature of MWSSR system due to certain steel wire degradation. The experimental setup for wire rope dynamics investigations, wire rope sample characteristics, testing schedule, and theoretical basics for signals processing are fully detailed within Section 2. The results yielded for tree significant cases of artificially induced damages within the rope structure are presented in Section 3. The results are individually analyzed and comparatively discussed within Section 4. Section 5 contains a few concluding remarks that try to underline the relevance of proposed methodology in this study.

## 2. Experimental Setup, Materials and Methods 

### 2.1. Experimental Setup Description

**Figure 1:**
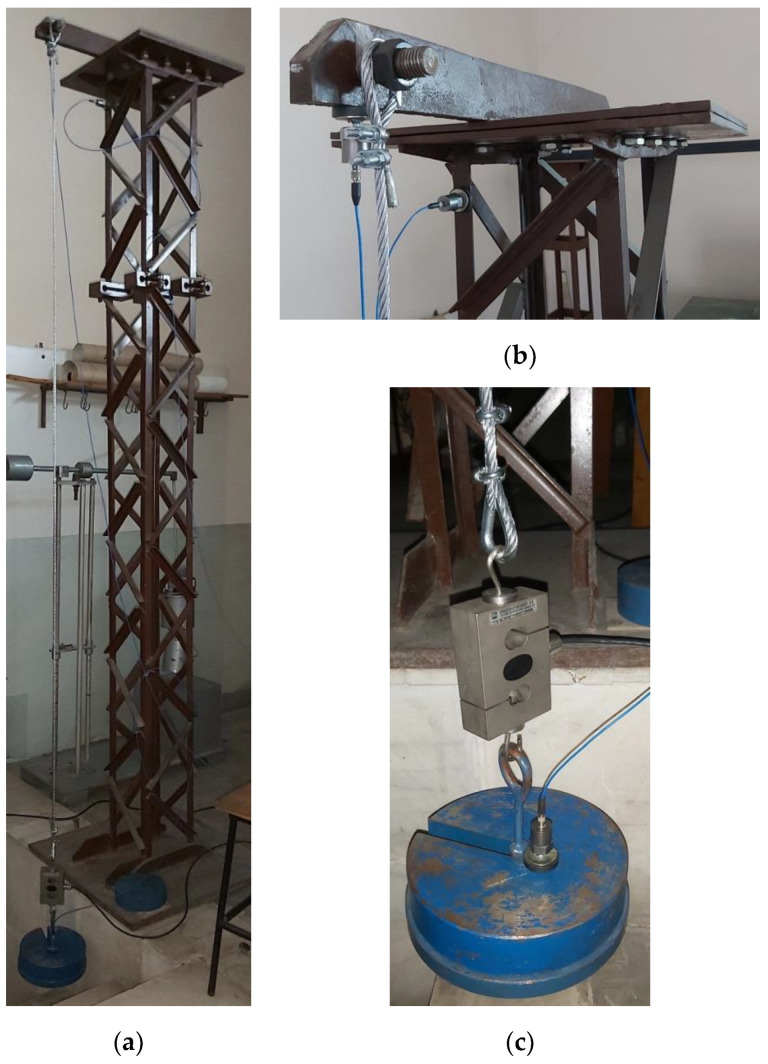
The experimental setup: (**a**) full view of laboratory stand; (**b**) detail view of rigid tower upper side device for hanging the wire rope, with parasite motions monitoring transducers; and (**c**) detailed view of loading mass ensemble, with force and acceleration transducers.

Experimental setup used for laboratory investigative tests contains a rigid latticed tower, which sustains, on vertical direction, the wire rope samples charged by static loads (see Figure 1). Dynamics of the MWSSR-based oscillating system was investigated using acceleration and force transducers. The transducers were mounted such that they enable monitoring the oscillations of loading mass, the parasite vibration of the upper side of support tower, and the force within the MWSSR sample. Images within Figure 1 present a full view of the laboratory stand (Figure 1a), as well as detailed views for upper hanging point of the rope on tower device (Figure 1b) and the static loading mass ensemble (Figure 1c). The transducer montages are also shown in detailed views in Figure 1.

**Figure 2:**
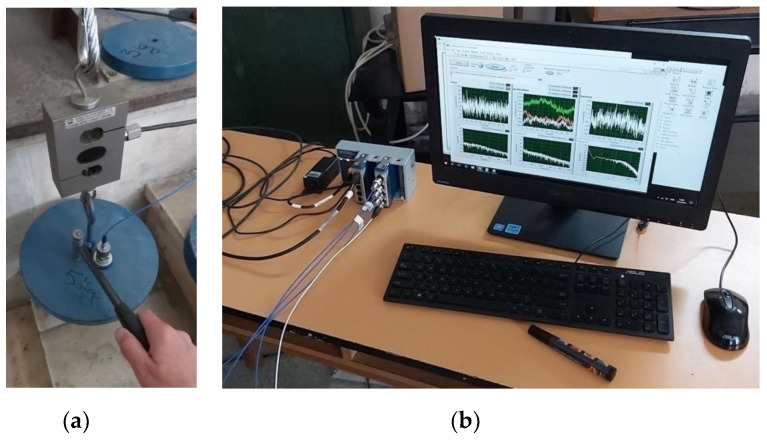
Details regarding testing procedure: (**a**) the scheme of initial impact pulse application using the impact hammer; and (**b**) ensemble view of digital acquisition system and computer with data management application.

Dynamic test was carried out using a mechanical shock, which was applied to the hanged mass (static load) to induce vibratory regime into the wire rope ensemble. An impact hammer supplied dynamic perturbation to the wire rope ensemble, which could provide an output signal proportional with the applied impact force (see Figure 2a). The initial transitory regime of the wire rope and mass ensemble was acquired and analyzed.

All signals provided by transducers (including the impact hammer output) were synchronously acquired with the help of the cDAQ-9174© chassis (National Instruments, Austin, TX, USA), supplied with digital acquisition boards: NI-USB-9233© (National Instruments, Austin, TX, USA) for accelerometers and NI-USB-9237© (National Instruments, Austin, TX, USA) for the strain-bridge based force transducers (see Figure 2b). Preliminary processing and data management were supplied by an application developed within LabVIEW software (National Instruments, Austin, TX, USA). Post-processing and analyses were performed with dedicated applications developed in Matlab software (MathWorks, Natick, MA, USA).

### 2.2. MWSSR Samples and Experimental Testing Cases

**Figure 3:**
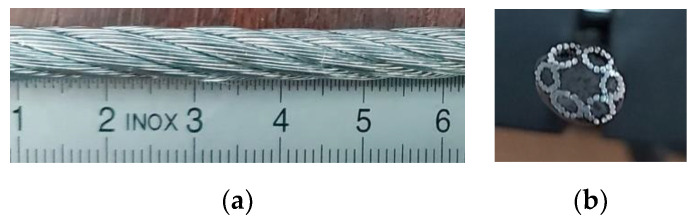
Wire rope subjected to dynamic experimental tests: (**a**) lateral view with rule for geometrical characterization; and (**b**) transversal sectional view.

The strands wire rope considered in this analysis was a regular 6(12+*FC*) + *FC* commercial cable, type CA1AA072A© (CABLERO, Iasi, Romania), 6-mm diameter, based on 6 strands with 12 steel wires of 0.4-mm diameter, designed for 288-kg maximum service load (traction load) for utilization on anchorage and kinematical applications, without any flexural loads due to pulley-based devices (Figure 3). The symbol *FC* within its code means that it has a fiber core, both for stands and for the rope. The unitary length mass is 0.11 kg/m and the theoretical failure load is 9.82 kN [12]. The nominal metallic cross-section area *S*, evaluated with the expression (π/4)*fd*^2^, acquires the value *S* = 9.05 mm^2^, where *d* denotes cable diameter, and the fill factor *f* yields 0.32 value, according to the steel wire and whole cable diameters ratio δ/*d* = 0.4/6 = 0.067 [12].

The experimental schedule considered many tests, with both initial integrity cable samples and artificially induced damages. Each sample had 2.200 m length between linkage devices. All samples were adopted from the same wire rope. Pre-tensioning force was enabled by a static load with mass *m* = 19 kg.

The defects produced during the use of strands wire rope within technical systems include wire breakage, wear, deformation, rust, and fatigue. Among them, fatigue includes various representations: internal and external cracks, internal and external wire breakages, and slack. In practice, the above damages interact with each other. Among these, the wire breakage is the main cause of MWSSR failure. It will reduce the strength and increase the potential safety hazards of MWSSR [11]. Thus, we exclusively considered the structural damage case of steel wire breakage.

**Figure 4:**
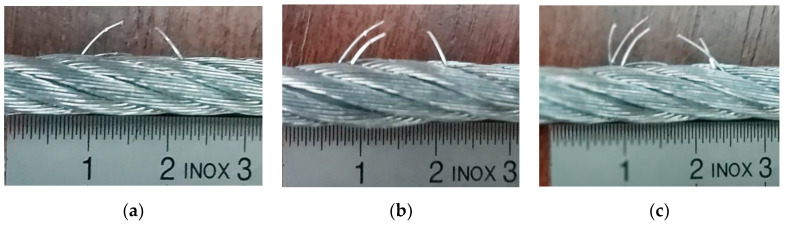
Artificial damages within wire rope structure adopted for presentation: (**a**) damage assigned to second case; (**b**) damage assigned to third case; and (**c**) damage assigned to fourth case. Note that the first case was considered as full integrity (undamaged inspected cable).

The damage induced into the cable structure during the tests consist of an artificial rupture of steel wire. It was considered a damage succession of one by one to three wires within a single rope strand. Taking into account the objective of this paper, the scenario of experiment was adopted based on the hypotheses: (1) The method is able to identify dynamic effects due to one broken wire; (2) The method is able to identify spectral differences between one-step broken wires; (3) The experimental investigations start with the case of no damaged cable sample. Thus, the increasing of broken wires number (beyond three) is not necessary because it does not provide additional information, but only increases the number of iterations for post-processing procedure. The authors considered that the adopted experimental schedule could provide enough evidence to demonstrate the feasibility of the proposed techniques. Figure 4 shows the selected cases for presentation, with specification that the first case supposed undamaged inspected cable (strands rope with full integrity). 

The same procedure of dynamic response evaluation of the rope-mass ensemble was carried out for each case and situation of analysis. To facilitate further comparative analysis of results, a computational procedure intended for timed harmonization of acquired signals was used. Effectively, it was used 0.1 s until the impact, 3.0 s time length of signal, and 10^4^ samples/s acquisition rate, for all signals, cases, and situations. For high-order spectral analyses (see next section), short time-length signals of 0.05 s were adopted, cutting up from 0.005 s until the impact moment. To avoid the residual noise within high frequency spectrum, the signals from force transducers were initially filtered with a low-pass filter (*f_cut_* = 400 Hz) within the acquiring equipment (analog filtering). 

### 2.3. Theoretical Basics for Signals Post-Processing and Investigations

The assessments within this study were formulated based on the hypothesis that the inspected wire rope and the loading mass formed a vibrating system, and the dynamic response due to experimental/operational perturbations contains essential information about the wire rope characteristics. Obviously, due to the changes during the exploitation time, these are able to provide useful information regarding potential structural/functional damage.

Considering the monitored residual motions of the hanging point on the upper side of the latticed support tower, the post-processing procedure primarily implied the evaluation of the absolute acceleration of loading mass, through rejection of parasitic components from the main acceleration acquired on the loading mass. Note that the vertical direction was considered acceleration (along the cable) to simplify the post-processing and analysis computational steps.

We considered that changes between analyzed cases would be more evident on spectral composition of investigated signal. Thus, we took three categories of spectral methods into account, starting from the simplest to the advanced (related to their intrinsic capability of analysis). It was assumed that constant scale methods based on Fourier transform (of both low and high order) provide basic information, while multi-scale techniques carry out deep data investigations. 

Acquired signals were investigated, in terms of spectrum composition, in three main directions: (i) using classical Fourier transforms and cross-spectral techniques; (ii) using high-order spectral analysis techniques; and (iii) using complex multi-scale methods, mainly based on wavelet algorithms. Theoretical basics and computational formulations for all directions are briefly presented in this section.

The first direction of analysis contains the evaluation of spectral composition for each acquired signal. A Fourier Transform-based computational algorithm, known as Discrete Fourier Transform (DFT), was used, and the results were exclusively considered in terms of magnitude. Supposing an input data sequence $\left\{x_{n}\right\}=x_{0},x_{1},x_{2},\ldots ,x_{N-1}$, the DFT algorithm will transform it into another sequence $\left\{X_{n}\right\}=X_{0},X_{1},X_{2},\ldots ,X_{N-1}$ with the expression [13,14]

(1) $$X_{k}=\sum_{n=0}^{N-1}x_{n}\;e^{-\frac{i2\pi }{N}kn}=\sum_{n=0}^{N-1}x_{n}\;\left(\cos\left(\frac{2\pi }{N}kn\right)-i\;\sin\left(\frac{2\pi }{N}kn\right)\right)$$

The spectra of the impact hammer force and the loading mass displacement directly result in the transfer function of the investigated system. Since the impact force and the displacement were both acquired at the same location (situated onto loading mass), the evaluated transfer function denotes the driving point function, which is the system admittance as viewed through the input point

(2) $$H\left(f\right)=\frac{1}{{\left(2\pi f\right)}^{2}}\frac{a\left(f\right)}{F\left(f\right)}$$

where *H* is the driving point transfer function, *f* denotes the frequency, and *a* and *F* represent acceleration and force spectra, respectively [14,15].

Supposing that the investigated ensemble respects the single-input–single-output (SISO) system schematization, the coherence between the input and the output was evaluated for each case, using the expression of magnitude-squared coherence *C_xy_*(*f*) [16]

(3) $$C_{xy}\left(f\right)=\frac{{\left|G_{xy}\left(f\right)\right|}^{2}}{G_{xx}\left(f\right)\;G_{yy}\left(f\right)}$$

where *G_xy_*(*f*) is the cross-spectral density between input/output investigated signals *x* and *y*, respectively *G_xx_*(*f*) and *G_yy_*(*f*) denote the auto-spectral density of the two signals.

A procedure involving the evaluation of coherence losing points in diagrams, followed by a comparative analysis may become time-consuming. In addition, this approach may produce results affected by errors due to the adopted technique of picking peaks. Hereby, the authors proposed a new approach, according which the coherence between initial and actual response of the monitored system is permanently computed. The responses are considered in terms of effective acceleration.

The second direction, involving high-order spectral analysis (HOSA) techniques, was adopted to increase the ability to detect fine changes within the response signal spectrum, where classical Fast Fourier Transform (FFT) might fail in proper evaluation. From the available methods, the authors selected the Wigner–Ville distribution (WVD) and the bi-spectrum (BS) evaluation.

The motivation for the WVD was that it offers excellent resolution in both the frequency and time domains, in contrast to the short time Fourier transform that has resolution limited in either time or frequency (related to window function) and suffers from smearing and side lobes leakage. Additionally, the Wigner function reduces the spectral density function at all times for stationary processes and is equivalent to the non-stationary autocorrelation function. Therefore, it roughly shows how the spectral density changes in time [17,18].

Supposing a signal *x*(*t*), the continuous WVD is given by [17,18]

(4) $$W_{x}\left(t,f\right)=\int_{-\infty }^{\infty }x\left(t+\frac{\tau }{2}\right)\;x^{*}\left(t-\frac{\tau }{2}\right)\;e^{-2\pi \;j\;\tau \;f}d\tau$$

where *t* and *f* denote time and frequency, respectively, and stared superscript indicates the conjugate. In the case of a unique signal *x*(*t*), the WVD is a bilinear function of the signal. 

Since the present study treats the problem of changes in spectral distribution of multiple signals (acquired on the same point, but at different moment of time), the authors used the WVD between the initial case and one of the other cases (successively considered). Additionally, in practice, the signals were usually provided by sampling procedures, thus a discrete formulation of WVD becomes necessary. The most utilized expression for discrete WVD, for two signals *x*(*t*) and *y*(*t*), is [17,18]

(5) $$W_{xy}\left(n,f\right)=\sum_{m}R_{xy}\left(m,n\right)\;e^{-j\;\pi \;f\;m}$$

with the instantaneous cross-correlation *R_xy_* defined by

(6) $$R_{xy}\left(m,n\right)=x^{*}\left(n-m\right)\;y\left(n+m\right)$$

where *n* is a parameter identified with time and *m* with lag. Note that the input signals must be sampled at twice the Nyquist rate or faster, in order to avoid aliasing.

To reject the cross-interference terms that result from the components that differ in both time and frequency center, a kernel function based on Choi–Williams distribution function was used. This windowing function, also known as exponential kernel function, can be calculated as [19,20]

(7) $$\Phi \left(\eta ,\tau \right)=e^{-\alpha {\left(\eta \tau \right)}^{2}}$$

where *η* and *τ* can be identified with frequency and time variables, respectively, and α represents the scale factor of the Choi-Williams window. This scale factor, usually constant, assures the suppression performance of the cross-terms (the smallest α values implies the better rejection performance) [19,20]. Thus, considering the inverse Fourier transform of this kernel function [19,20,21], and windowing the instantaneous cross-correlation, the practical expression of complex WVD scaled by Choi–Williams window is

(8) $$C_{xy}\left(t,f\right)=\int \int \sqrt{\frac{\alpha }{4\pi \tau^{2}}}\;e^{\frac{-\alpha t^{2}}{4\tau^{2}}}x\left(\mu +\frac{\tau }{2}\right)\;y^{*}\left(\mu -\frac{\tau }{2}\right)\;e^{-2\pi \;j\;\tau \;f}d\mu \;d\tau$$

BS ist he second HOSA technique used in the post-processing stage, because it can find nonlinear interactions [22]. In fact, BS is able to estimate the proportion of signal energy at any bi-frequency that enables quadratic phase coupling. BS is usually estimated as [22]

(9) $$B\left(f_{1},f_{2}\right)=F^{*}\left(f_{1}+f_{2}\right)\cdot F\left(f_{1}\right)\cdot F\left(f_{2}\right)$$

where *B* is the bi-spectrum evaluated at any frequencies *f*_1_ and *f*_2_, *F* denotes the Fourier transform of original signal at specified frequencies, and superscript symbol (*) indicates the complex conjugate. It has to be noted that the prefix “bi” in BS terminology refers not to two signals, but rather to two frequencies of a single signal. One additional variant for analysis may consist in bi-spectral coherency (simply known as bi-coherence), which represents a squared normalized version of BS. 

This polyspectral method is recommended to be used in the case of nonlinear interactions of a continuous spectrum of propagating waves in one dimension [21,22], thus that it may become very interesting in wire rope dynamic behavior investigation, especially for quantifying the extent of phase coupling in a signal.

Within present study, the authors performed comparative analysis between the BS of initial case and, successively, of every other case. Correlative estimations were performed in terms of cross-correlation method (with correlation coefficient and associated *p*-value outputs) applied to the two inspected BS. The correlation was separately evaluated for each frequency axis, and finally the results were averaged. This approach was adopted because the algorithm evaluates correlation coefficient and *p*-values for the correspondent column within the two investigated matrices. Thus, a pertinent evaluation of full correlation imposed a linear combination of results gained for each frequency axis.

The last direction of analysis supposes a complex multi-scale method for signal decomposition consisting of complex wavelet coherence (CWC) and cross-spectrum (CWCS). The HOSA techniques were involved to increase the changes estimation in spectral composition, while the multi-scale methods were additionally considered to gain certain significant spectral changes, but inoperable for the other algorithms.

**Figure 5:**
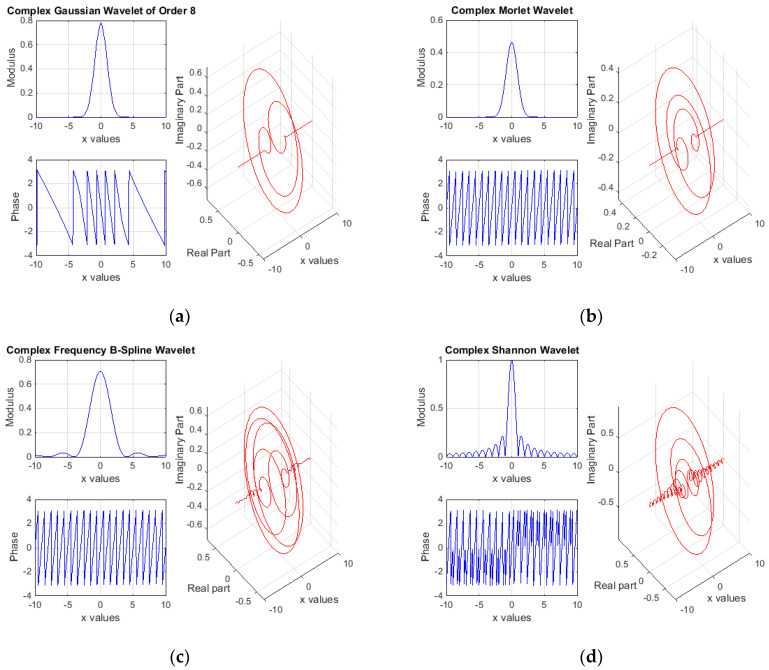
Wavelets for complex multi-scale analyses, in terms of modulus-phase components and real-imaginary parts respectively: (**a**) complex Gaussian wavelet of order 8; (**b**) complex Morlet wavelet; (**c**) complex frequency B-Spline wavelet; and (**d**) complex Shannon wavelet.

Initial wavelet-based estimations supposed four complex wavelet types, briefly presented as follows. Starting from the complex Gaussian function [23,24,25]

(10) $$f\left(x\right)=C_{p}\;e^{-jx}\;e^{-x^{2}}$$

results the complex Gaussian wavelet, by taking the *p*th derivative of function *f*. The integer *p* denotes the parameter of this wavelet family and in the previous formula, *C_p_* is such that $\left\|f^{\left(p\right)}\right\|=1$, where *f*^(*p*)^ represents the *p*th derivative of *f*. A graphical representation of complex Gaussian wavelet, in terms of both modulus-phase and real-imaginary parts, is depicted in Figure 5a.

The second type was the complex Morlet wavelet, defined by [23,24,25]

(11) ψ(x)=1π fbej 2 π fc x ex2fb

where the two parameters are the bandwidth *f_b_* and the wavelet center frequency *f_c_*. A graphical representation of complex Morlet wavelet (modulus-phase and real-imaginary parts) is depicted in Figure 5b.

Third type was the complex frequency B-spline wavelet, defined by [23,24,25]

(12) $$\psi \left(x\right)=\sqrt{f_{b}}{\left[\sin c\left(\frac{f_{b}x}{m}\right)\right]}^{m}e^{j\;2\;\pi \;f_{c}\;x}$$

and depending on three main parameters as follows. *f_b_* and *f_c_* have the same signification as previously mentioned and *m* is an integer-order parameter (m ≥ 1). Diagrams of complex frequency B-spline wavelet (modulus-phase and real-imaginary parts) are depicted in Figure 5c.

Last wavelet type was the complex Shannon wavelet, usually obtained from the frequency B-spline wavelet by setting unitary *m* [23,24,25]

(13) $$\psi \left(x\right)=\sqrt{f_{b}}\sin c\left(f_{b}x\right)\;e^{j\;2\;\pi \;f_{c}\;x}$$

Such that it depends on the same two parameters *f_b_* and *f_c_*. Figure 5d depicts the modulus-phase and real-imaginary parts of complex Shannon wavelet.

The continuous wavelet transform (CWT) was considered in terms of [23,24,25]

(14) $$W_{\psi }\left[f\right]\left(a,b\right)=W_{\psi }^{f}\left(a,b\right)=\left(f,\psi_{a,b}\right)=\int_{-\infty }^{\infty }f\left(t\right)\;\psi_{a,b}\left(t\right)dt$$

where the mother wavelet was defined [23,24,25]

(15) $$\psi_{a,b}\left(t\right)=\frac{1}{\sqrt{a}}\psi^{*}\left(\frac{b-t}{a}\right)$$

with *a* denoting the scaling parameter and *b* the translation parameter. In Equation (15), *Ψ* denotes the basic wavelet function and superscript symbol (*) indicates the complex conjugate. The wavelet function *Ψ* within the wavelet transform $W_{\psi }^{f}\left(a,b\right)$ must have zero mean and be localized in both time and frequency domain [24]. The wavelet is stretched in time by varying its scale *a* [25].

The authors adopted the complex Morlet wavelet to be used practically within this study, because it is able to provide a good balance between time and frequency localization [24,25].

The wavelet-based investigations used the complex wavelet cross-spectrum, defined as the product by CWT of first signal *f* with the complex conjugate CWT of second signal *g* [25]

(16) $$W_{\psi }^{fg}\left(a,b\right)=W_{\psi }^{f}\left(a,b\right)\cdot W_{\psi }^{g}{}^{*}\left(a,b\right)$$

The wavelet coherence results as a normalized wavelet cross-spectrum [25]

(17) $$C_{\psi }^{fg}\left(a,b\right)=\frac{{\left|W_{\psi }^{fg}\left(a,b\right)\right|}^{2}}{{\left|W_{\psi }^{f}\left(a,b\right)\right|}^{2}{\left|W_{\psi }^{g}\left(a,b\right)\right|}^{2}}$$

Since inspected signals were provided by an initial sampling procedure, the wavelet transform in Equation (14) has to be reconsidered for discrete data application. Hereby, the discrete wavelet transform (DWT) of a time series (*x_n_*, *n* = 1, …, *N*) with uniform time steps *δt* is defined as the convolution of *x_n_* with the scaled and normalized wavelet [25]

(18) $$W_{\psi }^{x}\left(a,b\right)=\sqrt{\frac{\delta t}{a}}\;\sum_{n=1}^{N}x_{n}\;\psi \left(\left(n-b\right)\frac{\delta t}{a}\right)$$

For clarity, the primarily used investigation techniques are summarized. In terms of particular changes between inspected cases (during the exploitation time), following aspects were considered:

(i) essential peaks in FFT magnitude of mass acceleration;
(ii) frequencies of losing coherency, both for each considered cases and between initial and successively actual cases;
(iii) consistent regions within initial transitory time domain on WVD diagrams;
(iv) shape changes and symmetry losing on cross-correlation between BS diagrams; and
(v) low values within the scale-time diagrams related to CWC between initial and successively actual cases.

## 3. Results

The particular aspects related to the five evaluation points presented in the last paragraph of Section 2 were followed in results post-processing and making of correspondent diagrams. 

**Figure 6:**
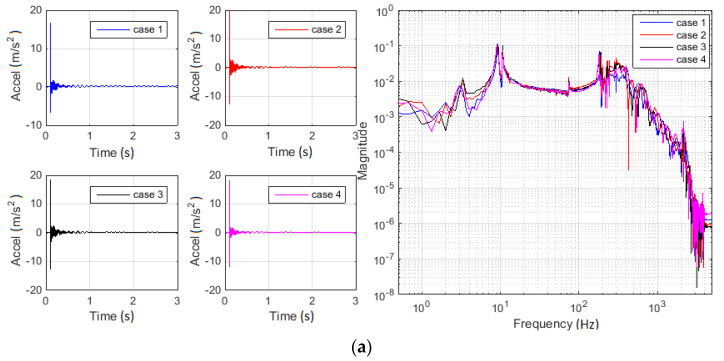

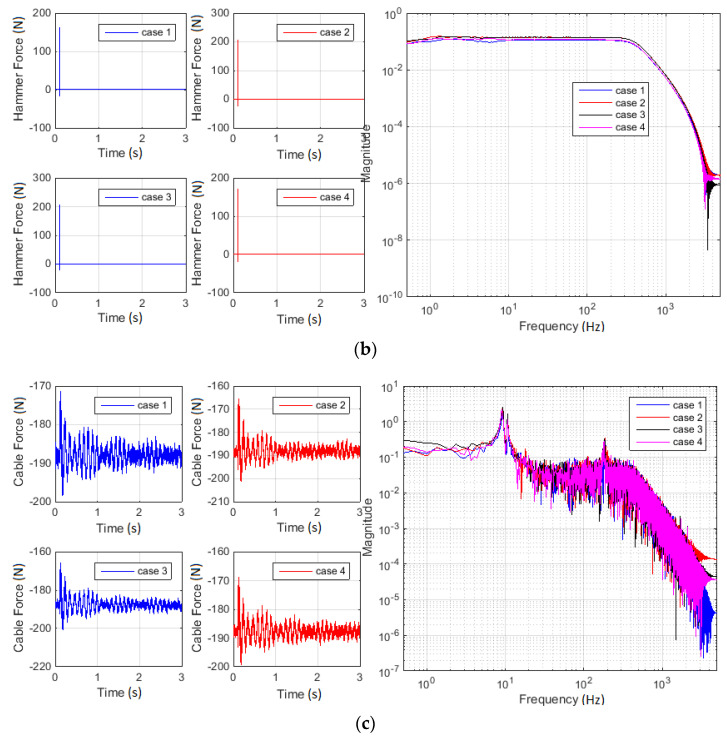
Dynamic perturbation and system responses for all analyzed cases: (**a**) effective acceleration of loading mass; (**b**) initial applied shock; and (**c**) force within the wire rope. Timed evolutions are separately presented, and FFT magnitudes are overlapped.

Firstly, the raw acquired signals are comparatively presented in Figure 6. The initial dynamic perturbation (in terms of applied shock) and the responses of the rope–mass ensemble (in terms of effective acceleration of loading mass and force within the rope) were considered. Figure 6 presents both separately timed signals and overlapped spectral compositions as FFT magnitudes.

**Figure 7:**
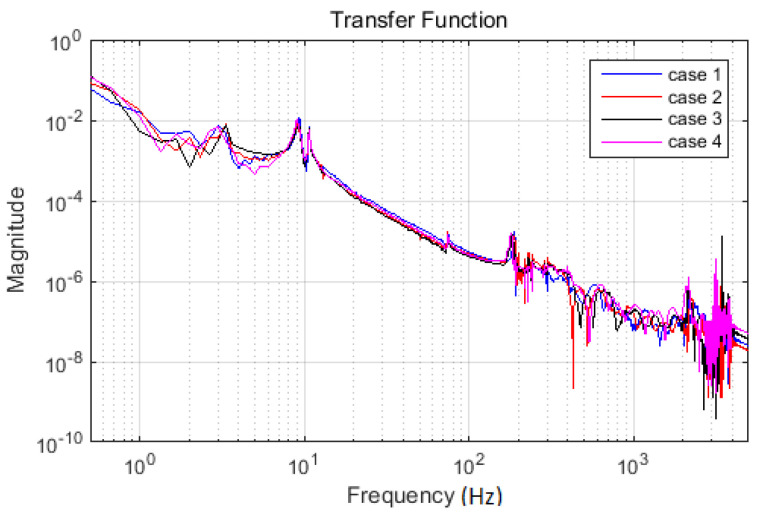
Driving point transfer functions for all analyzed cases. Overlapped presentation of FFT magnitudes is considered.

In addition, the driving point transfer function was evaluated for the investigated case. The results, in terms of magnitude, are overlapped in Figure 7.

**Figure 8:**
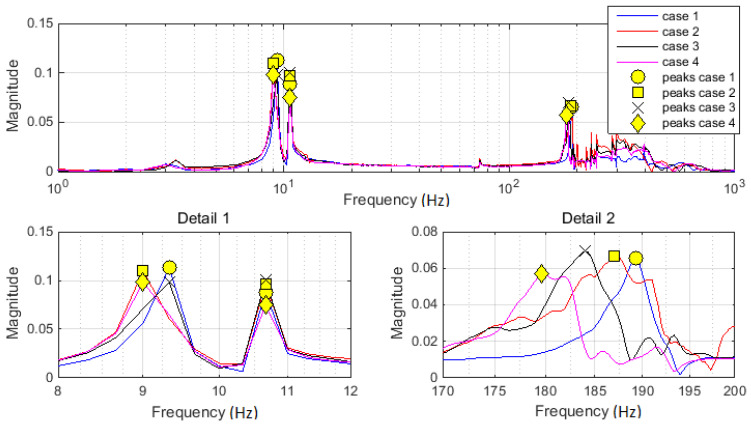
FFT magnitude of mass accelerations with marked significant peaks (in frequency range of interest). The first detail diagram focuses on the frequency range around 10 Hz. The second detail diagram focuses on the frequency range around 185 Hz. Markers and colors are explicated in the legend.

Relevant peaks within spectral diagrams were evaluated using a special computational procedure. It was considered the first five peaks, with the amplitude over 10% of maximum amplitude in spectrum, and the minimum prominence of 0.03 (constant values adopted based on the estimated results within the initial pre-processing stage). The respective values were suitably marked on spectra (see Figure 8). Two detailed views are additionally provided on Figure 8 to facilitate the analysis within the frequency ranges of interest. Effective values are detailed and discussed in the next section.

**Figure 9:**
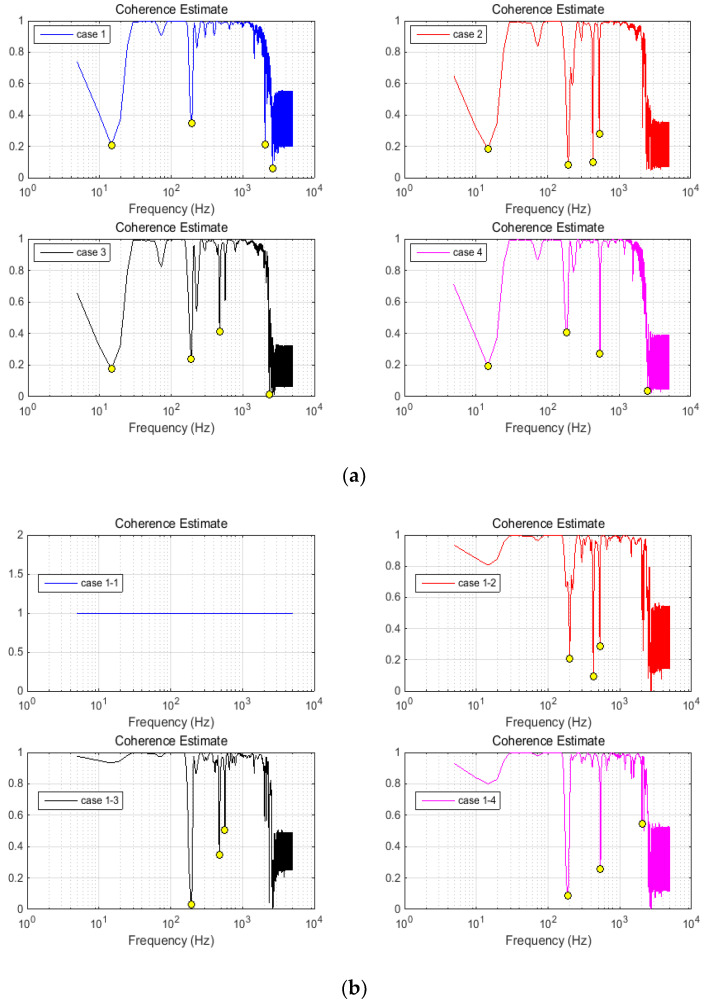
The coherence estimate of analyzed signals: (**a**) coherences between the input force and acceleration response for each case separately; and (**b**) coherences between the initial case and every other. Relevant minimum peaks are marked on diagrams.

The response coherences evaluated between acceleration and force signals are depicted in Figure 9a. The effective acceleration and the force within the wire rope were considered. The frequencies with coherency losses were evaluated and marked on each diagram (Figure 9a). In addition, the coherence between the initial case and every other was successively estimated. The results are depicted in Figure 9b, where diagrams also contain the frequencies related to coherency losses. These values were picked to facilitate comparative analysis and are discussed in the next section.

**Figure 10:**
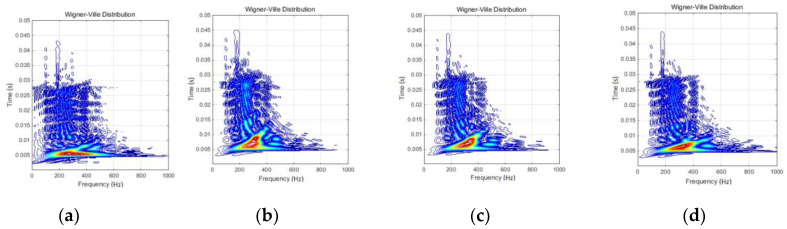
WVD of the rope - mass ensemble response in terms of loading mass effective acceleration: (**a**) Case 1; (**b**) Case 2; (**c**) Case 3; and (**d**) Case 4.

**Figure 11:**
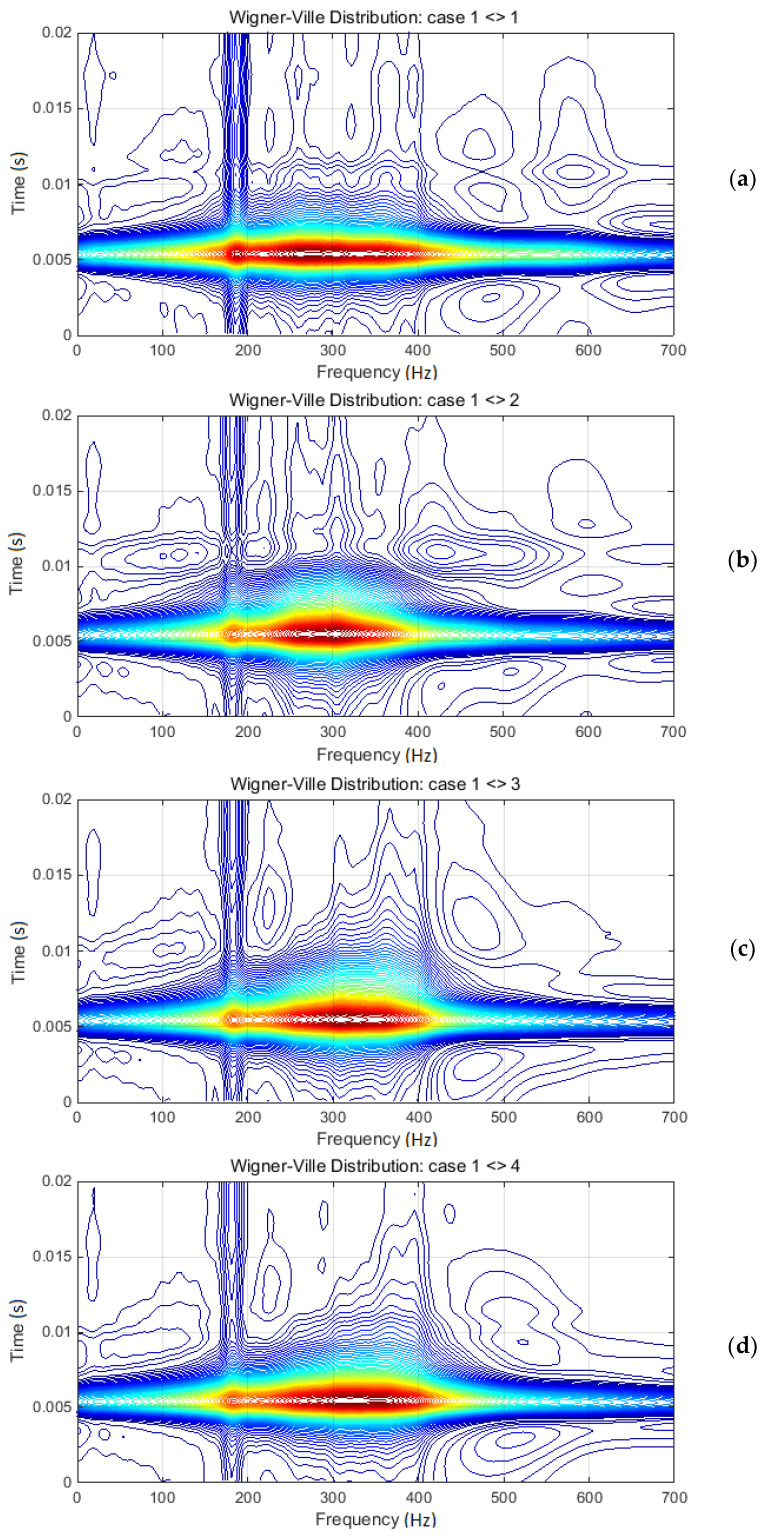
Cross-WVD diagrams: (**a**) Case 1 related itself; (**b**) Case 2 related to Case 1; (**c**) Case 3 related to Case 1; and (**d**) Case 4 related to Case 1. Diagrams are focused on time–frequency relevant domain.

Figure 10 presents a set of joint time–frequency analysis, based on WVD applied on each acceleration response. Because of the certain subjectivity in comparing of changes between these diagrams, the cross-WVD of each damage cases with the initial case was additionally evaluated. The results are presented in Figure 11. The first case in Figure 11 is related to cross-WVD of the initial case itself.

**Figure 12:**
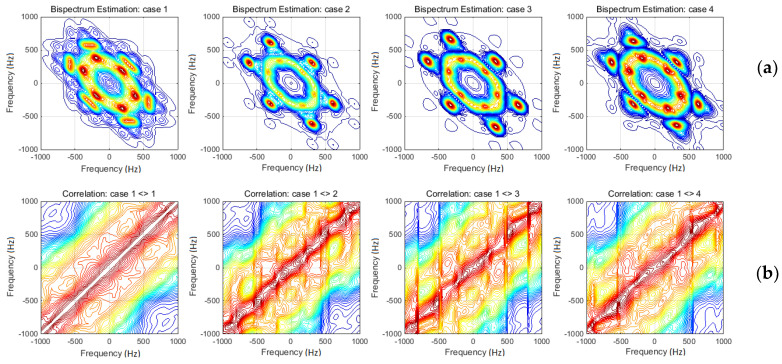
Bi-spectral analyses: (**a**) bi-spectrum diagrams for each considered cases; and (**b**) correlation coefficients between the first case and every other.

**Figure 13:**
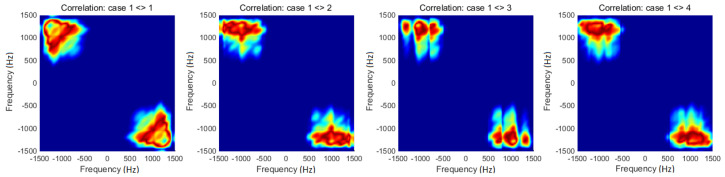
The *p*-values related to the correlation coefficients in Figure 12b. Extended frequency ranges are adopted to show relevant values at domain extremes.

The results of HOSA based on BS technique are depicted in Figure 12a. Respective diagrams contain the response acceleration BS for each investigated case. Comparative analyses between the four diagrams might be affected by certain subjectivity. Hereby, a unique and objective method was necessary. The authors adopted the correlation method, successively applied between the initial case and every other. The primary results, in terms of correlation coefficients, are presented in Figure 12b. The *p*-values related to these coefficients were also estimated, and the results are presented in Figure 13.

**Figure 14:**
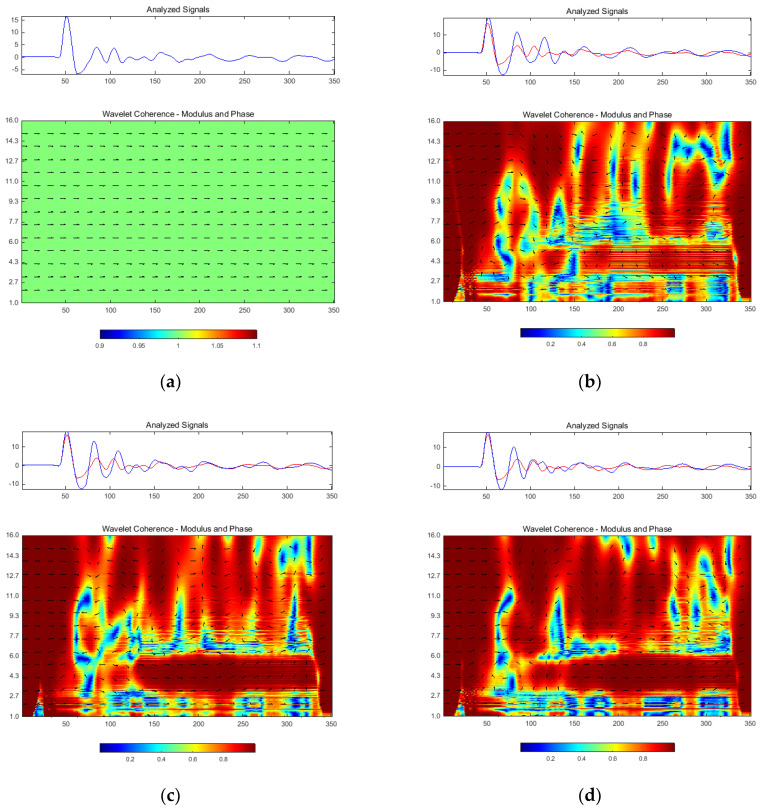
Modulus of complex wavelet coherence with arrow-map overlapped phase information: (**a**) Case 1 related itself; (**b**) Case 2 related to Case 1; (**c**) Case 3 related to Case 1; and (**d**) Case 4 related to Case 1. Each pair of inspected signals is also depicted. Horizontal axis denotes instant sample number. Vertical axis of CWC indicates the wavelet scale.

**Figure 15:**
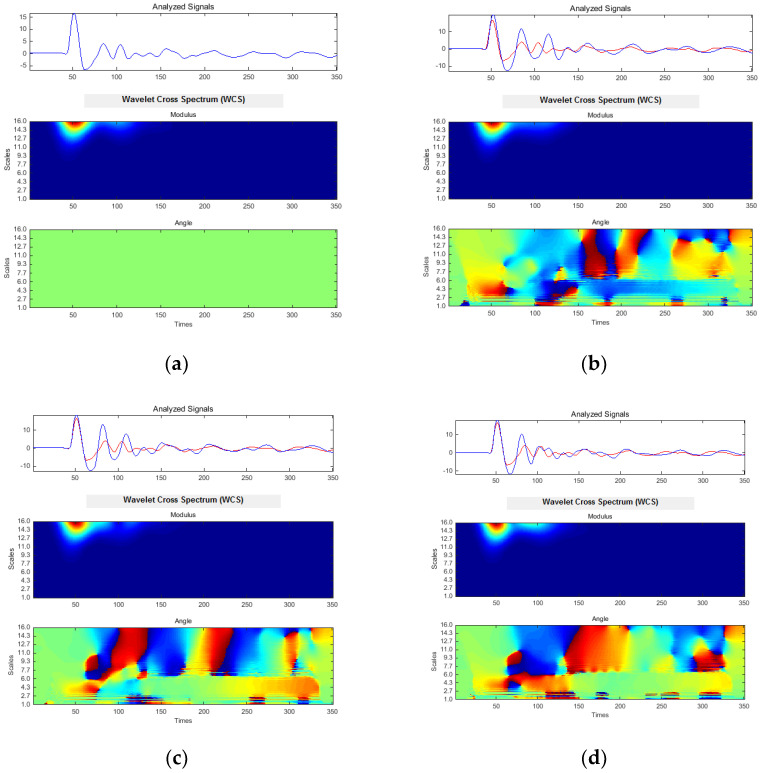
Modulus and phase of complex wavelet cross-spectrum: (**a**) Case 1 related itself; (**b**) Case 2 related to Case 1; (**c**) Case 3 related to Case 1; and (**d**) Case 4 related to Case 1. Each pair of inspected signals is also depicted. Horizontal axes denote instant sample number.

The results obtained in the last stage of response signals post-processing supposed a multi-scale method based on wavelet transform. CWT of each response signal was computed. CWC and CWCS were also evaluated for each pair (acceleration-force) of response signals. Following the real difficulties met in comparison procedure, the authors again adopted the previously used working hypothesis: the estimation of CWC/CWCS between initial case and every other. The results in terms of CWC are presented in Figure 14, and in terms of CWCS in Figure 15. The phase information mapped on the coherence graph (see Figure 14) and modulus–angle pair (see Figure 15) were also considered.

## 4. Discussion

The analysis and discussions follow each stage of the signal post-processing. Comparative analysis of raw signals diagrams in frequency domain (FFT magnitudes in Figure 6) showed a good correlation between investigated cases related to the essential peaks positions. Spectral diagrams also revealed the frequency ranges of interest. The entire study was based on the characterization of potential changes within these domains and the relation with the inspected cases.

Relevant peaks were picked both from the acceleration spectra (Figure 6) and from the driving point functions (Figure 7). The frequencies related to these peaks were identically valued for both categories of spectra. These values are presented in Table 1. Considering the clearness of graphical presentation, the acceleration FFT magnitude is marked in Figure 8. The purpose of these diagrams results from the necessity to estimate the frequency range, where the changes due to the artificially imposed damages were sought.

Differences between values in Table 1 already showed a strong relation of damage case with the dominant peak changes. It should be mentioned that the first peak is related to the latticed tower. It had no changes, thus it provided irrelevant information within this study.

Following the results within previous section, let us go on to comparative analysis of coherency losses. The first step supposed the response pair coherences (see Figure 9a). The second step considered the cross coherence between the testing cases (see Figure 9b). Both steps provided interesting information about the changes of drop-down frequencies. The values related to the first step are presented in Table 2 and those of the second step in Table 3. Compared to the FFT magnitude, the coherence additionally supplied a few relevant frequencies. Simultaneously following these frequencies within their changes can provide more precise evaluation of the rope damage.

The previous ensemble of analysis techniques can supply periodic monitoring and estimations of ropes structural health with feasible results. Therefore, continuously monitoring, which is especially applied on operational conditioning, may become insensible due to the inherent dynamic perturbations that can also induce spectral components. These components can be easily confounded with those essentially related to the potential damage.

HOSA specific techniques are able to provide enough distinctive peaks related to the signal spectrum. Thus, it would be more facile to identify the frequency of interest and to follow the changes during inspection procedure. WVD of response accelerations (see Figure 10) showed the changes between investigated cases more accurately. Damage evolution induced certain segregation of relevant lobes in the time–frequency domain of interest. 

The comparative analysis revealed the time scale enlarging of “red zone” in respect to damage evolution; at the same time, it was systematically stretched along the frequency scale during the inspection time. Case 1, of undamaged rope, presented a local transitory regime, corresponding to the perturbation moment and covering the range of 150–450 Hz. The rope damage (Case 2) induced a strength reduction of frequency coverage at impact time (under 400 Hz), but an obviously shifting tendency in time (during the 5 ms after the impact moment). Analyzing Cases 3 and 4 resulted that this initial tendency slowly decreases in respect to the damage amplification, but maintains reduced coverage at impact moment. A partial conclusion was that the damage evolution reduces the initial frequency spreading but extends it in time scale.

However, the procedure following these changes, especially for many cases, might become inaccurate due to the multiple peaks within the transitory domain. Thus, as previously assumed, the cross-WVD between specific cases can help the estimation of relevant changes. Figure 11 shows the results of cross-WVD estimated between the initial case and every other. Comparative analysis revealed the more accurate tendency of maximum peaks segregation due to damage evolution, while also clearly shifting the tendency during time scale. It can be observed that one relevant peak decreases very slowly under 200 Hz, and the other peak clearly shifts from 270 Hz (according to undamaged case), through 300 Hz (according the first damage case), to 355 Hz (according to last damage case). Obviously, the diagrams in Figure 11 are focused on the time–frequency domain of interest, taking into account the maximum values. Nevertheless, the analysis of less relevant, but clearly represented, areas within the diagrams might reveal supplementary information regarding the linkage between damage evolution and changes in spectral distribution.

The second HOSA technique used by the authors was the BS of response accelerations (see Figure 12a). Considering the symmetry provided by these diagrams, the analysis may be conducted only into the two adjacent quadrants. Note that the first quadrant-based analysis is not able to provide additional information to the FFT results. The segregation of spectral lobes and the setting off and the shifting tendency of the relevant peaks were clearly shown by the BS. Finding relevant peaks and following them during the evolution of rope damage form a simple way to accurately evaluate the situation. In addition, the authors used the correlation algorithm to more accurately evaluate the changes between each diagram on their evolution. The correlation coefficients (averaged between successive estimations according the two frequency axes) are presented in Figure 12b. Case 1 (of undamaged wire rope) presents an evident symmetry related to the first bisector. The correlations between Case 1 and every other one reveal a certain loss of this symmetry. The changes become more evident to the damage evolution, due to the segregation of dominant lobes according to the horizontal axis. The extreme domains (those related to positive–negative frequency pairs) also become sources of additional information about damage evolution. Extending the frequency ranges and considering the *p*-values associated to the correlation coefficients (see Figure 13), the changes between BS diagrams become more evident in terms of frequency lobes segregation and scattering.

Multi-scale spectral analysis was involved in this study to provide, not more accurate results than previously presented, but better investigations of each spectral range due to variable scale of inspection. Complex Morlet wavelet type was adopted due to its advantages (see Section 2). CWC and CWCS algorithms were supposed for correlative multi-scale investigations between experimental cases. Since the CWC diagrams in Figure 14 were augmented with phase information related to CWCS angle evolutions (see Figure 15), these results are discussed entirely. It is evident that coherence modulus presents relevance on its smallest values (near to zero), thus the evolution of dark blue areas shown in Figure 14 were followed. 

The analysis was primarily focused on the domain along the scale axis, nearly after the samples corresponding to the initial perturbation (the initial dynamic impact applied onto the loading mass). Each wavelet-based diagram was accompanied by the timed analyzed signals (in terms of samples actual number). 

Hereby, the domain of interest was analyzed within the 50–150 times (samples) values. Scales between 1 and 16 were adopted, which means a range from 351 to 21.94 Hz for considered wavelet type. If on HOSA based diagrams a certain segregation tendency with the damage evolution were observed, the CWC presented a clear tendency of grouping and airing the minima values into investigated domain. The CWC in Figure 14b (Cases 1 and 2) presents minima at scales 1–2.7, 3, 4.3–6, and 9. The next case shown in Figure 14c indicated incipient grouping tendency at scales 1.5, 2.7–4.3, and 6–11, together with a size reduction of minima zones along the samples axis. Grouping tendency had become more evident for the last case (Figure 14d), where a single predominant zone was clearly contoured between 2.7 and 11 scale values. Additionally, a second group was observed for 1–2.7 scales, which was increasingly stretched, along the inspected time domain, in respect to the damage evolution cases. The tendencies identified on CWC diagrams in Figure 14 are more evident on CWCS angle diagrams within Figure 15 following the sudden phase changes (for time scale between 50 and 150 samples). The general tendency of CWCS phase is to change its directivity, from time axis according to minor damage case, to scale axis according to the damage increasing.

## 5. Conclusions

Following the remarks above, a minimum set of experiments is enough for identification of potential damage within wire rope. The changes in spectral composition of response signals, especially the effective acceleration of loading mass, can be provided by multiple spectral investigation techniques, starting with simple FFT analysis, by coherence algorithms, to advanced HOSA and wavelet-based methods. Each of them can provide accurate information according to specific aspects such as experimental or operational testing procedure, number of successive available cases related to the damage evolution, the instantaneous magnitude of rope damage, and initial availability of data related to a proper reference case. Regarding the last aspect, it has to be noted that the initial reference case of no rope damage is recommended to be adopted. However, the proposed investigation procedure, for any of the presented methods, can be applied using any other case as the reference, but respecting the damage–time evolution. This means that the reference case must be taken as the first case from the available succession of testing cases.

The limitation of this research consists in exclusively using of experimental data, without any operational mode acquired signals. This study demonstrated the ability of the proposed technique to carry out the identification of damages within wire rope elements through a suitable post-processing methodology. The apparent limitation on experimental investigation will be treated in a future work, which will provide additional findings based on operational dynamic tests procedure.

Future developments of proposed technique will be focused on suitable computational applications able to provide more accurate results for the situations implying low rate of signal–noise ratio (especially for operational tests). HOSA and wavelet-based algorithms will surely supply these applications because of their certain intrinsic capability to ignore noise effects.

## Figures and Tables

**Table 1:** Frequency values related to the essential peaks in FFT diagrams.

Case Number	Frequency (Hz)			
	1st Peak	2nd Peak	3rd Peak	4th Peak
Case 1	9.3330	10.6663	189.3270	–
Case 2	8.9997	10.6663	186.9938	299.3234
Case 3	9.3330	10.6663	183.9939	–
Case 4	8.9997	10.6663	179.6607	–

**Table 2:** Frequency values related to coherency losses for each inspected case.

Case Number	Frequency (Hz)			
	1st Peak	2nd Peak	3rd Peak	4th Peak
Case 1	14.6484	195.3125	2036.1328	2617.1875
Case 2	14.6484	195.3125	429.6875	527.3438
Case 3	14.6484	190.4297	473.6328	2329.1016
Case 4	14.6484	185.5469	537.1094	2490.2344

**Table 3:** Frequency values related to coherency losses between reference and every inspected case.

Case Number	Frequency (Hz)		
	1st Peak	2nd Peak	3rd Peak
Case 1-2	200.2	429.7	527.3
Case 1-3	195.3	473.6	566.4
Case 1-4	190.4	–	537.1
